# Loss of CDKL5 Causes Synaptic GABAergic Defects That Can Be Restored with the Neuroactive Steroid Pregnenolone-Methyl-Ether

**DOI:** 10.3390/ijms24010068

**Published:** 2022-12-21

**Authors:** Roberta De Rosa, Serena Valastro, Clara Cambria, Isabella Barbiero, Carolina Puricelli, Marco Tramarin, Silvia Randi, Massimiliano Bianchi, Flavia Antonucci, Charlotte Kilstrup-Nielsen

**Affiliations:** 1 Department of Biotechnology and Life Sciences (DBSV), Centre of NeuroScience, University of Insubria, 21052 Busto Arsizio, Italy; 2 Department of Medical Biotechnology and Translational Medicine (BIOMETRA), University of Milan, 20129 Milan, Italy; 3 Ulysses Neuroscience Ltd., Trinity College Dublin, D02 PN40 Dublin, Ireland; 4 Institute of Neuroscience, Trinity College Dublin, D02 PN40 Dublin, Ireland

**Keywords:** CDKL5, inhibitory synapse, gephyrin, collybistin, GABAA receptor, pregnenolone-methyl-ether

## Abstract

CDKL5 deficiency disorder (CDD) is an X-linked neurodevelopmental disorder characterised by early-onset drug-resistant epilepsy and impaired cognitive and motor skills. CDD is caused by mutations in cyclin-dependent kinase-like 5 (CDKL5), which plays a well-known role in regulating excitatory neurotransmission, while its effect on neuronal inhibition has been poorly investigated. We explored the potential role of CDKL5 in the inhibitory compartment in *Cdkl5*-KO male mice and primary hippocampal neurons and found that CDKL5 interacts with gephyrin and collybistin, two crucial organisers of the inhibitory postsynaptic sites. Through molecular and electrophysiological approaches, we demonstrated that CDKL5 loss causes a reduced number of gephyrin puncta and surface exposed γ_2_ subunit-containing GABA_A_ receptors, impacting the frequency of miniature inhibitory postsynaptic currents, which we ascribe to a postsynaptic function of CDKL5. In line with previous data showing that CDKL5 loss impacts microtubule (MT) dynamics, we showed that treatment with pregnenolone-methyl-ether (PME), which promotes MT dynamics, rescues the above defects. The impact of CDKL5 deficiency on inhibitory neurotransmission might explain the presence of drug-resistant epilepsy and cognitive defects in CDD patients. Moreover, our results may pave the way for drug-based therapies that could bypass the need for CDKL5 and provide effective therapeutic strategies for CDD patients.

## 1. Introduction

Mutations in the X-linked cyclin-dependent kinase-like 5 (*CDKL5*) gene cause a severe neurodevelopmental disorder (Early Infantile Epileptic Encephalopahty, OMIM 300672), commonly referred to as CDKL5 deficiency disorder (CDD). CDD patients are characterised by intellectual disability, autistic features and drug-resistant epilepsy that normally manifest within the first three months of age [1]. *CDKL5* is mutated in approximately 1:50.000 live births, making CDD one of the most frequent causes of genetic epilepsy.

CDKL5 is a serine-threonine kinase that is highly abundant in the brain and whose expression peaks in the first postnatal weeks [2]. The constitutive loss of CDKL5 in CDD mouse models causes impaired learning and memory, altered locomotion and autistic-like features [3,4,5,6]. Spontaneous epilepsy has only recently been observed in aged heterozygous female mice [7,8] and in male mice harbouring the conditional knock-out (KO) of *Cdkl5* in glutamatergic forebrain neurons [9].

Various studies of *Cdkl5* mouse models and CDKL5 deficient primary neurons converge on the role of CDKL5 in regulating excitatory neurotransmission [6]. Indeed, CDKL5 interacts with the scaffolding protein PSD95, and CDKL5 deficient neurons are characterised by morphological and molecular alterations linked to excitatory synapses [10,11,12]. These defects depend in part on altered microtubule (MT) dynamics leading to the impaired invasion of MTs into dendritic spines and reduced spine maturation. Indeed, we previously demonstrated that CDKL5 regulates the MT-binding of the plus-end tracking protein CLIP170, thus impacting MT dynamics [13,14,15]. Importantly, the modulation of MT dynamics in vitro and in vivo, mediated by treatment with the pregnenolone analogue pregnenolone-methyl-ether (PME), which promotes CLIP170 functioning, can restore several CDKL5-related defects, including spine maturation, expression of synaptic proteins and hippocampal-dependent learning and memory [13,14,15].

Notwithstanding the importance of maintaining a proper excitation/inhibition balance, the possible role of CDKL5 in regulating inhibitory neurotransmission has ‘til now been rather neglected. Mice with the conditional inactivation of *Cdkl5* in their glutamatergic forebrain neurons display altered miniature inhibitory synaptic currents (mIPSCs) [16], but a molecular basis for such a defect is still unknown.

Here, we report that CDKL5 interacts with the inhibitory postsynaptic scaffolding complex containing gephyrin and collybistin (CB) and show that CDKL5 loss leads to reduced levels and functioning of the γ_2_ subunit-containing γ-aminobutyric acid type A receptors (GABA_A_R) in vitro and in vivo. GABA_A_Rs are heteropentameric Cl^−^ permeable channels, generally composed of two α- and two β-subunits and a single γ- or δ-subunit [17]. Typically, synaptic GABA_A_Rs, which mediate phasic inhibition upon presynaptic GABA release, contain a γ-subunit; contrariwise, the extrasynaptic receptor complexes that mediate tonic inhibition as a response to low ambient GABA levels contain the δ-subunit [18]. Surface levels of synaptic GABA_A_Rs depend on a complex and dynamic regulation including transport, recycling and stabilisation, of which the latter relies on a scaffolding complex containing gephyrin and CB [19].

Alterations in any of the components linked to inhibitory neurotransmission are associated with various neurodevelopmental disorders characterised by cognitive defects, autism-like features and epilepsy [20]. The pharmacological targeting of the GABAergic system, based both on the allosteric modulation of GABA_A_R subtypes and on the functional control of GABA_A_R-associated proteins, represents an important interventional strategy against epilepsy and other clinical manifestations linked to the altered GABA_A_R levels or functioning [21].

We here show that treatment with PME can restore GABA_A_R γ_2_ expression and functioning in primary *Cdkl5*-KO neurons. Importantly, we also find that the synaptic GABA_A_R subunit γ_2_ is significantly reduced in hippocampi of *Cdkl5*-KO mice, but that its levels are normalised to those of WT animals upon treatment with PME. Altogether, these data suggest that CDKL5, through its interaction with the inhibitory scaffolding complex, regulates synaptic GABA_A_R levels and, importantly, that altered membrane insertion of GABA_A_Rs can be restored by the targeting of MT dynamics.

## 2. Results

### 2.1. CDKL5 Interacts with the Gephyrin-Collybistin Complex and Regulates the Number of Postsynaptic Gephyrin Clusters

**Figure 1:**
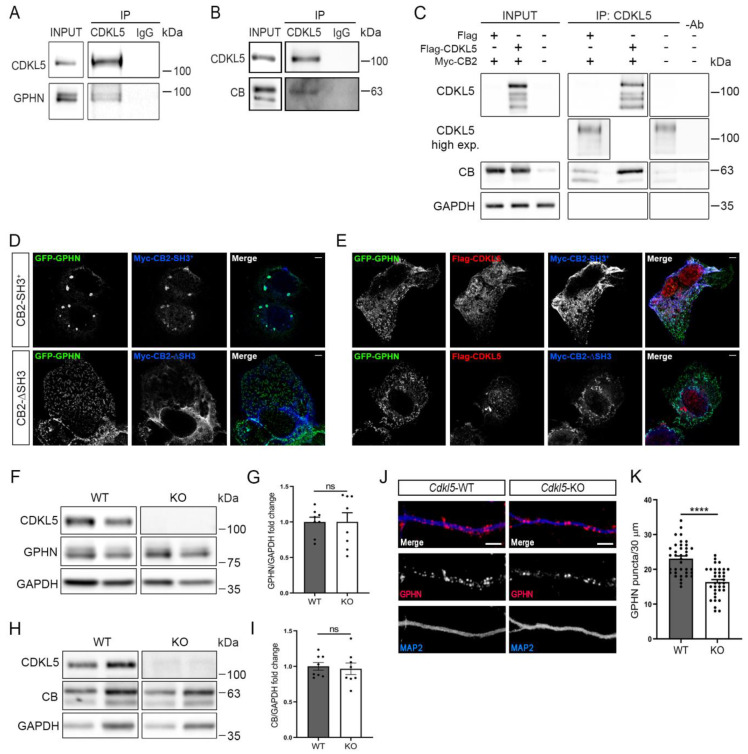
CDKL5 interacts with the gephyrin-collybistin complex. (**A**,**B**) Representative WBs showing the coimmuniprecipitation of CDKL5 and gephyrin (GPHN; A) or collybistin (CB; B) from PND20-30 mouse brain lysates. Unrelated IgGs were used as negative control. Whole brain lysate (input; 2.5%) and immunocomplexes were analysed through WB with antibodies against CDKL5, GPHN (**A**) and CB (**B**). n = 3. (**C**) Flag-CDKL5 was expressed in HEK293T cells together with Myc-CB2 and immunoprecipitated with a monoclonal anti-CDKL5 antibody. Whole cell lysates (input; 3%) and immunoprecipitated proteins were analysed through WB using antibodies against CDKL5, CB and, as loading control, GAPDH. n = 3. (**D**,**E**) Representative confocal images of COS7 cells expressing GFP-tagged gephyrin (GFP-GPHN, green) and Myc-CB2-SH3^+^ or Myc-CB2-∆SH3 (blue) together with Flag-CDKL5 (red). Scale bar: 5 µm. (**F**,**H**) Representative WB of whole cell lysates of *Cdkl5*-WT/KO primary hippocampal neurons at DIV14. Antibodies against GPHN and CB were used together with anti-CDKL5 and, as loading control, GAPDH. (**G**,**I**) The graphs show the quantification of normalised GPHN (**G**) and CB (**I**) levels. n = 8 biological replicates. Mean ± SEM. Not significant (ns), *p* > 0.05. Unpaired Student’s *t*-test. (**J**) Representative images of DIV14 *Cdkl5*-WT/KO hippocampal neurons stained with antibodies against GPHN (red) and the dendritic marker MAP2 (blue). Scale bar, 5 μm. (**K**) Graph showing the quantification of GPHN puncta along 30 μm long segments. n = 7 biological replicates, N = 37/35 WT/KO neurons. Mean ± SEM. **** *p* < 0.0001. Unpaired Student’s *t*-test.

To investigate the possible role of CDKL5 at the inhibitory synapses, we examined its interaction with gephyrin and CB, two proteins playing a fundamental role in the organisation of the postsynaptic sites of these synapses. To this aim, brain lysates of young mice at postnatal day 20–30 (PND20-30) were used to immunoprecipitate CDKL5 using IgGs as negative control; through the subsequent western blotting (WB), both gephyrin and CB could be detected in the CDKL5 immunocomplexes (Figure 1A,B). We further confirmed the interaction in a heterologous system expressing Flag-tagged CDKL5 in HEK293T cells together with the Myc-tagged CB2 isoform (Figure 1C). Upon immunoprecipitation of CDKL5, allowing the precipitation of both exogenous and endogenous CDKL5, overexpressed CB2 could easily be detected through WB analysis. Interestingly, the weakly expressed endogenous CB could also be detected as interacting with endogenous CDKL5 that was visible upon a higher exposure of the membrane. These results confirm a recent report by Uezu et al. [22] in which CB was identified as a direct interactor of CDKL5 through a chemical-genetic proximity-labelling approach.

Gephyrin is a key protein in the organisation of inhibitory synapses through the formation of submembranous clusters that regulate the accumulation of GABA_A_Rs at the postsynaptic sites [23]. The accumulation of gephyrin under the cell membrane depends on the guanine nucleotide exchange factor CB that can recruit gephyrin to the postsynaptic sites. CB exists in a closed inactive conformation, which depends on its N-terminal SH3 domain [24]. When GFP-tagged gephyrin is expressed together with the SH3-containing CB2 derivative (CB2-SH3^+^) in a heterologous system, it therefore accumulates in cytoplasmic deposits (Figure 1D), whereas deletion of the SH3 domain (CB-∆SH3) renders CB constitutively active, leading to the translocation of gephyrin to submembranous microaggregates. Interestingly, the exogenous expression of Flag-tagged CDKL5 in cells expressing CB2-SH3^+^ causes GFP-gephyrin to accumulate under the cell membrane, suggesting that CDKL5 is capable of activating CB through the interaction of the two proteins (Figure 1E).

We further evaluated whether CDKL5 loss influences the total levels of gephyrin or CB in neurons. WB analyses on neuronal lysates from primary hippocampal cultures of *Cdkl5*-WT and -KO neurons, cultured for 14 days in vitro (DIV), did not reveal any changes in the expression of either gephyrin or CB (Figure 1F–I). We next examined whether CDKL5 loss affects the capacity of gephyrin to form the typical submembranous clusters in primary hippocampal neurons. Interestingly, we observed a significantly reduced number of gephyrin puncta in the dendritic segments of *Cdkl5*-KO neurons (Figure 1J,K). The interaction of CDKL5 with the gephyrin-CB complex, together with the reduced number of gephyrin clusters in *Cdkl5*-KO neurons, indicate that CDKL5 might play a hitherto undescribed role in the organisation of the postsynaptic sites of inhibitory synapses.

### 2.2. CDKL5 Loss Affects the Membrane Levels of γ_2_ Subunit-Containing GABA_A_Rs and Impairs mIPSCs

**Figure 2:**
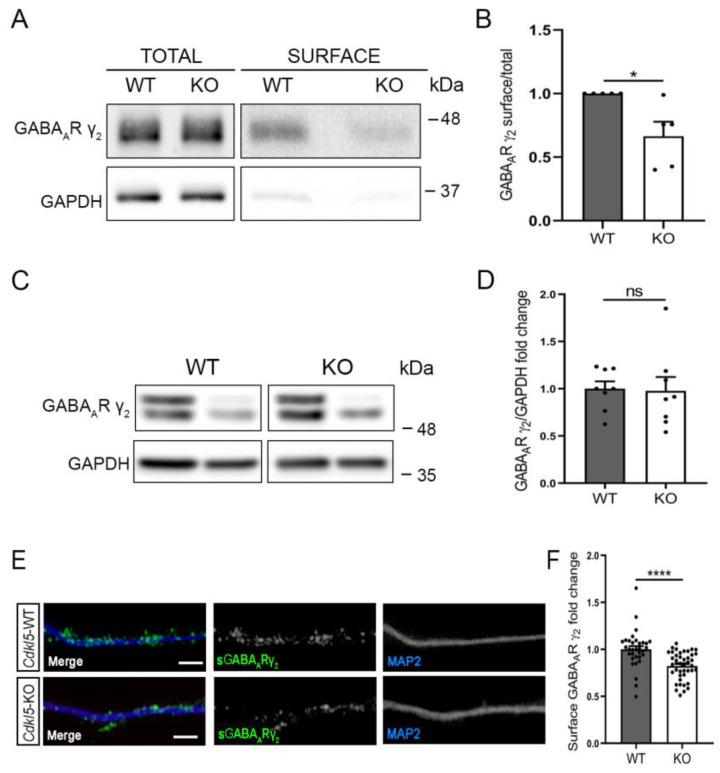
CDKL5 loss affects surface expression of synaptic GABA_A_Rs in primary hippocampal neurons. (**A**) Representative WB of a biotinylation experiment on *Cdkl5*-WT/KO primary hippocampal neurons at DIV14. Levels of GABA_A_R subunit γ_2_ were analysed together with GAPDH in the surface fraction, obtained from 500 µg of lysate, and in 30 µg of whole cell lysate. (**B**) Graph showing the ratio of surface/total levels of GABA_A_R γ_2_. N = 5 biological replicates. Mean ± SEM * *p* < 0.05. Unpaired Student’s *t*-test. (**C**) Representative WB analysis of whole cell lysates of *Cdkl5*-WT/KO primary hippocampal neurons at DIV14. GAPDH was used as loading control. (**D**) Graph showing the quantification of normalised GABA_A_R γ_2_ levels. N = 8 biological replicates. Mean ± SEM ns, *p* > 0.05. Unpaired Student’s *t*-test. (**E**) Surface exposed GABA_A_R γ_2_ (green, sGABA_A_R γ_2_) was detected through immunostaining of *Cdkl5*-WT/KO primary hippocampal neurons at DIV14 under non-permeabilising conditions. MAP2 (blue) was used as dendritic marker. Scale bar, 5 µm. (**F**) Graph showing the quantification of the fluorescence intensity of sGABA_A_R γ_2_ staining of 30 µm long dendritic segments. n = 6 biological replicates, N = 33/42 WT/KO neurons. Mean ± SEM **** *p* < 0.0001. Unpaired Student’s *t*-test.

Gephyrin deficiency impacts the surface accumulation of γ_2_-containing GABA_A_Rs [25,26,27]. We therefore speculated that the reduced number of gephyrin clusters in *Cdkl5*-KO neurons might influence the cell-surface expression of the GABA_A_R subunit γ_2_. To address this, we performed a cell-surface biotinylation assay. Briefly, upon biotinylation of hippocampal neurons, labelled proteins were affinity purified, and the amount of the synaptic GABA_A_R subunit γ_2_ was analysed through WB in parallel with a fraction of the total cell extract (Figure 2A). The proportion of the total receptor pool that resides in the neuronal surface was determined by quantifying the ratio of the biotinylated fraction (surface) over the amount in the total lysate (total). As control of the biotinylation procedure, we verified that the cytoplasmic protein GAPDH was barely present in the pool of affinity-purified proteins. As illustrated in the graph in Figure 2B, we found that *Cdkl5*-KO neurons displayed reduced surface levels of the γ_2_ subunit, whereas its total levels were unaltered (Figure 2C,D). We further examined the surface expression of the γ_2_ subunit through the immunofluorescence staining of hippocampal *Cdkl5*-WT and -KO neurons performed under non-permeabilising conditions. In accordance with the biotinylation experiment, *Cdkl5*-KO neurons displayed significantly reduced surface levels of the γ_2_ subunit (Figure 2E,F).

**Figure 3:**
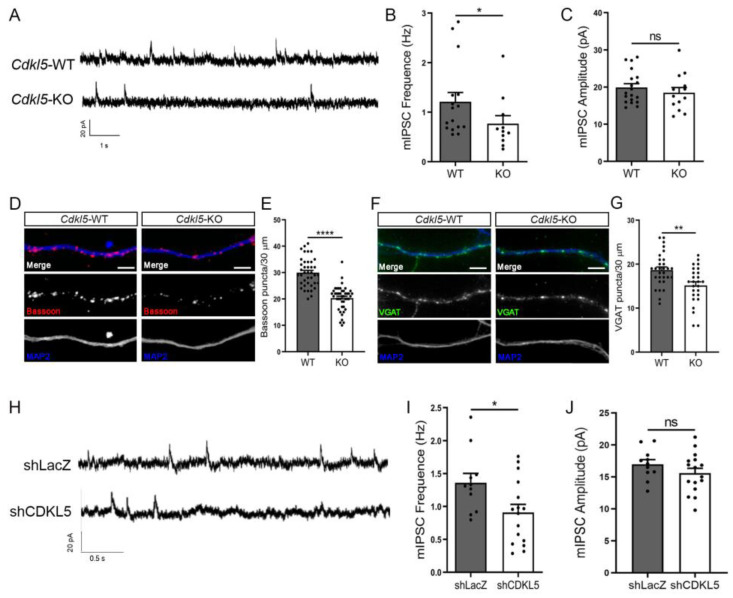
CDKL5 loss leads to a reduction in the frequency of mIPSCs and in presynaptic inhibitory markers. (**A**) Representative traces of mIPSCs recorded from *Cdkl5*-WT/KO primary hippocampal neurons at DIV14 patching at +10 mV. (**B**,**C**) Graphs showing mIPSC frequency (**B**) and amplitude (**C**) in *Cdkl5*-WT/KO cultures. n = 3 biological replicates; frequency: N = 16/11 WT/KO neurons. Mean ± SEM * *p* < 0.05, not significant (ns), *p* > 0.05. mIPSC frequency: Mann Whitney U test; mIPSC amplitude: Unpaired Student’s *t*-test. (**D**,**F**) Representative images of *Cdkl5*-WT/KO neurons at DIV14 stained for Bassoon (red, D), VGAT (green, F) and MAP2 (blue). Scale bar, 5 μm. (**E**,**G**) Graphs showing the quantification of bassoon and VGAT puncta along 30 μm long dendritic segments. Bassoon: n = 7 biological replicates, N = 40/44 neurons. VGAT: n = 3 biological replicates, N = 30/26 neurons. Mean ± SEM ** *p* < 0.01, **** *p* < 0.0001. Unpaired Student’s *t*-test. (**H**) Representative traces of mIPSCs recorded from CDKL5-silenced neurons (shCDKL5) and controls (shLacZ). Neurons were transfected with GFP-expressing shRNA vectors at DIV11 and mIPSCs were recorded at DIV14 patching at +10 mV. (**I**,**J**) Graphs showing mIPSC frequency (**I**) and amplitude (**J**) upon acute CDKL5-silencing. n = 3 biological replicates; N = 11/16 shLacZ/shCDKL5 neurons. Mean ± SEM * *p* < 0.05; ns, *p* > 0.05. Unpaired Student’s *t*-test.

To evaluate the functional consequences of the reduced levels of the γ_2_-containing GABA_A_Rs on the neuronal surface, we measured mIPSCs from *Cdkl5*-WT and -KO hippocampal neurons at DIV14. Notably, in *Cdkl5*-KO neurons, mIPSCs were less frequent, while the amplitude was unaltered (Figure 3A–C). The reduced frequency of mIPSCs points to possible presynaptic deficits in the *Cdkl5*-KO neurons, and we therefore analysed the presynaptic markers bassoon and vesicular GABA transporter (VGAT). Bassoon is localised at the active zone of both excitatory and inhibitory presynaptic terminals, while VGAT is specific for the inhibitory presynaptic sites. Immunofluorescence staining showed reduced numbers of both bassoon and VGAT puncta in *Cdkl5*-KO hippocampal neurons (Figure 3D–G).

The interaction of CDKL5 with the postsynaptic scaffolding proteins led us to test whether the ablation of CDKL5 at the postsynaptic site would be sufficient to generate the observed reduction in mIPSC frequency. We therefore recorded the mIPSCs in neurons transfected with a construct expressing a short-hairpin RNA specific for CDKL5 (shCDKL5) or, as control, against LacZ (shLacZ). Transfected neurons are easily detectable thanks to the concomitant expression of GFP from these vectors; moreover, due to the low transfection efficiency (below 5%), the synaptic input to transfected neurons is generated from non-silenced cells. Interestingly, the mIPSC frequency was reduced in shCDKL5 neurons as compared to the shLacZ controls (Figure 3H–J), thus supporting a postsynaptic CDKL5-dependent effect on presynaptic inputs.

### 2.3. GABAergic Defects in Cdkl5-KO Neurons Are Normalised upon Treatment with PME

**Figure 4:**
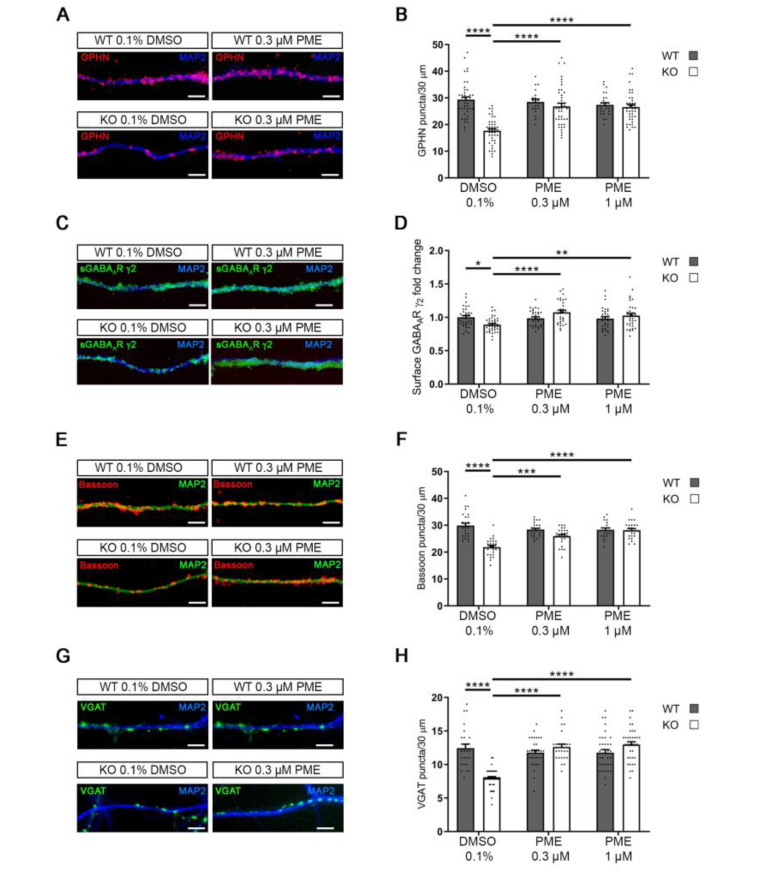

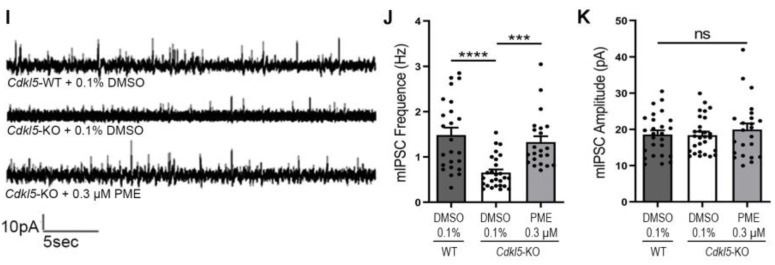
Treatment with PME normalises CDKL5-dependent inhibitory synaptic defects. (**A**,**C**,**E**,**G**) Representative images of *Cdkl5*-WT/KO primary hippocampal neurons stained for MAP2 together with gephyrin (GPHN, red; A), GABA_A_R γ_2_ (green, C), bassoon (red, E) or VGAT (green, G) upon treatment with 0.3 or 1 µM of PME or vehicle (0.1% DMSO) at DIV11 for 72 h. Scale bar, 5 µm. (**B**,**D**,**F**,**H**) Graphs showing the quantification of GPHN puncta (**B**), sGABA_A_R γ_2_ fluorescence intensity (**D**), bassoon puncta (**F**), VGAT puncta (**H**) along 30 µm long dendritic segments. GPHN: n = 6 biological replicates, N ≥ 19 neurons; sGABA_A_R γ_2_: n ≥ 5 biological replicates, N ≥ 32 neurons; bassoon: n = 3 biological replicates, N ≥ 20 neurons; VGAT: n = 3 biological replicates, N ≥ 25 neurons. Mean ± SEM * *p* < 0.05; ** *p* < 0.01; *** *p* < 0.001; **** *p* < 0.0001. Two-way ANOVA, followed by Tukey’s post-hoc. (**I**) Representative traces of mIPSCs in *Cdkl5*-WT and -KO primary hippocampal neurons treated as indicated with either vehicle (0.1% DMSO) or 0.3 µM PME for 72 h starting at DIV11. (**J**,**K**) Graphs showing mIPSC frequency (**J**) and amplitude (**K**) of *Cdkl5*-WT and -KO neurons treated as indicated. n = 3 biological replicates, N ≥ 22. Mean ± SEM. mIPSC frequency: *** *p* < 0.001, **** *p* < 0.0001. Kruskal-Wallis test, followed by Dunn’s multiple comparisons test. mIPSC amplitude: ns, *p* > 0.05. One-way ANOVA, followed by Tukey’s post-hoc.

The presence of GABA_A_Rs on the neuronal membrane is determined by the dynamic balance of delivery, recycling and degradation, which altogether depends on complex regulatory mechanisms involving the interaction of various proteins, including gephyrin, with MTs [28,29]. We previously showed that various neuronal defects linked to CDKL5 deficiency in vitro and in vivo can be restored through treatment with PME, which promotes MT dynamics [13,14,15]. We therefore found it intriguing to analyse whether the treatment of *Cdkl5*-KO neurons with PME could restore the observed defects at the inhibitory synapse. *Cdkl5*-WT and -KO neurons were treated with 0.3 and 1 µM PME at DIV11 and stained at DIV14 for gephyrin, GABA_A_R γ_2_, bassoon or VGAT (Figure 4A–H). Whereas treatment with PME affected neither of the pre- and postsynaptic markers in WT neurons, we observed a significant effect already with 0.3 µM of PME in *Cdkl5*-KO neurons. Indeed, at the postsynaptic site, both the gephyrin and the GABA_A_R γ_2_ levels were restored to those in WT neurons (Figure 4A–D); a similar positive effect was observed also with the presynaptic markers bassoon and VGAT (Figure 4E–H). In line with this, treatment with PME could also normalise the frequency of mIPSCs in *Cdkl5*-KO neurons, whereas no effect was observed on the amplitude (Figure 4I–K).

**Figure 5:**
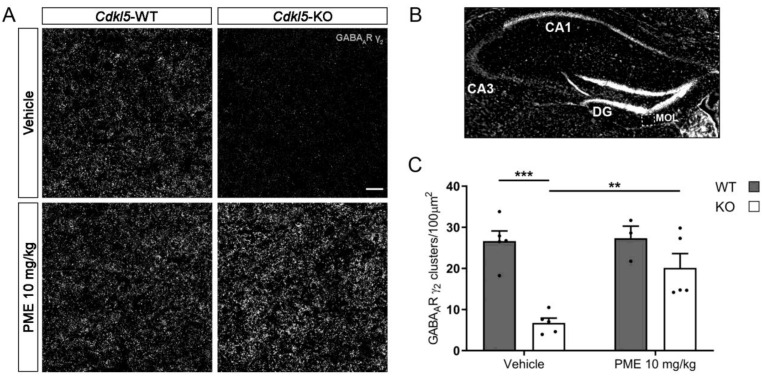
Reduced levels of synaptic GABA_A_R γ_2_ in hippocampi of *Cdkl5*-KO mice are normalised upon treatment with PME. (**A**) Hippocampal slices (dentate gyrus) from *Cdkl5*-WT/KO male mice treated with PME (10 mg/kg, s.c. for seven days starting from PND60) or sesame oil (vehicle) were stained for GABA_A_R γ_2_. Scale bar, 5 µm. (**B**) Representative image of a hippocampal section stained with the nuclear dye DAPI. Images from the molecular layer (MOL) of the dentate gyrus (DG) were used for the analyses shown in panels A and C. (**C**) Graph showing the quantification of GABA_A_R γ_2_ clusters/100 µm^2^. n ≥ 3. Mean±SEM, ** *p* < 0.01, *** *p* < 0.001. Two-way ANOVA followed by Tukey’s post-hoc.

By treating symptomatic *Cdkl5*-KO mice with PME, we previously found that CDKL5-related hippocampal-dependent behavioural and excitatory synaptic defects benefit from increased MT dynamics in vivo [15]. Intrigued by the positive effect of PME on inhibitory synapses in vitro, we proceeded to evaluate its effect in vivo, also. With this aim, we subjected hippocampal sections of *Cdkl5*-WT and -KO mice treated with 10 mg/kg of PME for 7 days (starting at PND60) to immunofluorescence staining against GABA_A_R γ_2_. As shown in Figure 5 we observed a dramatic decrease in the density of GABA_A_R γ_2_ clusters in the dentate gyrus of the vehicle-treated *Cdkl5*-KO mice with respect to the WT mice; interestingly, upon treatment with PME, the number of GABA_A_R γ_2_ clusters was similar between the two genotypes.

## 3. Discussion

In this study, we investigated the possible role of CDKL5 in the inhibitory synapse. Beyond the well-established role of CDKL5 at glutamatergic synapses, the results of this study reveal a hitherto undescribed function of CDKL5 in the inhibitory compartment. Our results suggest that CDKL5, through its interaction with the inhibitory scaffolding complex containing gephyrin and CB, regulates membrane levels of synaptic γ_2_-containing GABA_A_Rs. Deranged GABA_A_R levels are frequently linked to cognitive deficits and epilepsy, and we speculate that our results may help in explaining the seizure phenotype observed in CDD patients.

### 3.1. CDKL5 Deficiency Leads to Dysfunctions in the Inhibitory Synapse

GABA_A_Rs are ion channels permeable to chloride and bicarbonate ions and are, in the mammalian CNS, localised at postsynaptic inhibitory specialisations or at extrasynaptic sites, where they mediate inhibitory neurotransmission [29,30].

Herein, we analysed the synaptic GABA_A_Rs, focusing our attention on the γ_2_ subunit, which is the most abundant GABA_A_R subunit in the rat brain [17]. Our immunofluorescence analyses showed a significant reduction of GABA_A_R γ_2_ surface expression in both the hippocampal neurons and hippocampal slices of *Cdkl5*-KO mice. This defect was corroborated by biochemical approaches. Indeed, through the highly sensitive cell-surface biotinylation assay, we showed that the absence of CDKL5 influenced surface levels of γ_2_-containing GABA_A_Rs in primary cultures of *Cdkl5*-KO neurons. Since the absence of CDKL5 did not affect the total levels of the GABA_A_R γ_2_ subunit, the reduced surface levels might be a consequence of an altered transport or recycling.

The cell membrane distribution of synaptic GABA_A_Rs is dynamically regulated through various mechanisms, including the subsynaptic scaffolding factor gephyrin, which binds and clusters synaptic GABA_A_Rs at sites directly opposite to GABA-releasing axon terminals [23]. Through in vitro and ex vivo immunoprecipitation experiments, we found that CDKL5 forms a complex with both CB and gephyrin. Furthermore, the reduction of surface expressed synaptic GABA_A_Rs was accompanied by a reduction in the number of gephyrin-positive puncta in the *Cdkl5*-KO primary cultures.

The molecular interaction between CDKL5 and the cytoplasmic CB-gephyrin complex is likely a key mechanism through which CDKL5 exerts its control on synaptic GABA_A_Rs. In particular, gephyrin takes part in the aggregation, but not in the surface insertion or stabilisation, of α_2_ and γ_2_ subunit-containing GABA_A_Rs [31]. CB is a brain-specific GDP/GTP-exchange factor, which interacts with gephyrin and regulates its recruitment from intracellular deposits to postsynaptic membranes [32]. The loss of CB leads to a strong reduction in gephyrin and synaptic GABA_A_R clusters in several regions of the forebrain, including the hippocampus, amygdala and cerebellum [33,34]. Normally, CB is present in an auto-inhibited conformation and depends on other neuronal factors such as neuroligin 2 (NL2), GABA_A_R subunit α_2_ or the Rho-like GTPase TC-10 for its activation [35,36]. Our results, showing that exogenous CDKL5 expression is sufficient for localising gephyrin under the cell membrane when coexpressed with full-length CB, suggest that CDKL5 may also be capable of relieving CB from the inhibited conformation. Our data also support a previous study by Uezu et al. [22] that identified CDKL5 as a direct interactor of CB. The presence of gephyrin in the immunocomplexes is likely caused by an indirect interaction mediated by CB. Since CB is required for inhibitory receptor clustering and function, via the recruitment of gephyrin [37], we speculate that CDKL5 plays a direct role in the stabilisation of the key components of the inhibitory synapse by the above interaction. Future studies will be performed to address how CDKL5 loss affects the gephyrin-CB complex and which GABA_A_R subunits are affected.

### 3.2. CDKL5 Deficiency Impairs the Functional GABAergic Synapse

The postsynaptic inhibitory defects were accompanied by a reduction in the frequency of mIPSCs in *Cdkl5*-KO neurons. Various mechanisms can underlie the reduced frequency, such as reduced number of synapses at the pre- and postsynaptic levels, a reduced neurotransmitter release and a reduced number of presynaptic vesicles. Even if we cannot rule out a direct role for CDKL5 at the presynaptic level, we find it relevant to consider that a similar result was observed when CDKL5 expression was acutely silenced through the transfection of a CDKL5-silencing construct also expressing GFP. Our experimental settings allowed the recording of the mIPSCs of CDKL5-silenced neurons that were innervated by non-silenced cells. Therefore, the altered inhibitory neurotransmission could be ascribed to a direct role for CDKL5 at the postsynaptic site, given that the presynaptic compartment is normal. The major phenotype of CDKL5 deficiency in both *Cdkl5*-KO and silenced neurons was a remarkable decrease in mIPSC frequency, hence reflecting a strong reduction in the number of functional GABAergic synapses. We speculate that the loss of CDKL5 at the postsynaptic site influences GABAergic innervation in *Cdkl5*-KO cultures similar to what has been reported for γ_2_-containing GABA_A_R clusters in cortical neurons silenced for GODZ, which is implicated in trafficking and postsynaptic accumulation of γ_2_ subunit-containing GABA_A_Rs [38]. In support of our hypothesis, we observed a significantly reduced number of both bassoon- and VGAT-positive puncta in the primary cultures of *Cdkl5*-KO neurons, which indicates a presynaptic defect.

### 3.3. PME Treatment Ameliorates CDKL5-Related Defects

Various recent data have shown that CDKL5 is involved in regulating MT dynamics [13,39,40,41]. Interestingly, the possibility of targeting MT interacting proteins, the function of which is impaired in the absence of CDKL5, seems to represent an interesting disease modifying therapeutic strategy for CDD [14,15,42].

Here we show that treatment in vitro with the neuroactive synthetic steroid PME restores CDKL5-dependent GABA_A_R defects both molecularly and functionally. Indeed, the decreased frequency of mIPSCs in primary *Cdkl5*-KO cultures was normalised, together with the surface exposure of synaptic GABA_A_Rs and the number of gephyrin, bassoon and VGAT puncta. Intriguingly, we also found that the number of γ_2_ subunit-containing GABA_A_R clusters was restored in the hippocampal slices of *Cdkl5*-KO mice treated with subcutaneous injections of PME (10 mg/kg) for seven consecutive days starting from PND60. Of relevance, this treatment schedule restored hippocampal-dependent learning and memory defects in *Cdkl5*-KO mice [15].

At present, we can only speculate about the precise mechanism through which PME can promote synaptic GABA_A_R accumulation. Our previous data showed that PME activates the +TIP CLIP170 by inducing its open conformation, thus promoting MT dynamics [15]. CLIP170 was found to be involved in the efficient loading of dynein-bound cargoes for retrograde transport in axons [43]. Dynein is central for minus-end directed transport of various cargoes and, by activating CLIP170, PME may therefore promote dynein-dependent transport. Indeed, gephyrin interacts directly with dynein and transports glycine receptor-containing vesicles in dendrites [28]. Moreover, forward trafficking of γ_2_-containing GABA_A_Rs is mediated by GABARAP-containing complexes, which interact with dynein [44]. Future studies will allow us to reveal the effect of PME on dynein-dependent transport.

In conclusion, we have demonstrated for the first time that CDKL5 plays a direct role in the expression of functional GABA_A_Rs at synaptic sites, in part through its interaction with the cytoplasmic CB-gephyrin complex. Importantly, treatment with the synthetic neuroactive steroid PME can bypass the need for CDKL5 and restore GABA_A_R expression and GABA_A_R functioning. Currently, there are no approved therapies for CDD and any pharmacological strategies that reduce the frequency, duration or severity of seizures may positively impact the quality of life for CDD patients. PME might represent an important breakthrough in the CDD field, as the restoration of GABA_A_R expression might be beneficial also for the cognitive defects and autistic-like features present in CDD patients.

## 4. Materials and Methods

### 4.1. Mice

Protocols and use of animals were approved by the Animal Ethics Committee of the University of Insubria and in accordance with the guidelines released by the Italian Ministry of Health (D.L. 2014/26) and the European Community directives regulating animal research (2010/63/EU). Adult mice were euthanised by cervical dislocation, while neonates were sacrificed by exposure to CO_2_ followed by decapitation.

Male *Cdkl5*-KO mice [4] on the genetic background CD1 were used. Littermate controls were used for all experiments. The day of birth was designated as postnatal day (PND) zero. After weaning, three to five animals belonging to the same litter were housed in activity enriched cages on a 12 h light/dark cycle in a temperature-controlled environment with food and water provided *ad libitum* and checked daily for general health conditions. Genotyping was performed through PCR on genomic DNA from tail biopsies using the Rapid Extract PCR Kit (PCRBIO, [15]).

### 4.2. Plasmids

pEGFP-GPHN, pRK5 Myc-CB2-SH3^+^, and pRK5 Myc-CB2-∆SH3 were kindly provided by Dr. Theophilos Papadopoulos’s laboratory (Max-Planck Institute for Brain Research, Göttingen) and were generated as described previously [45]. pFlag-CDKL5 encoding the 107 kDa splice variant was generated as described elsewhere [46].

### 4.3. Cell Cultures and Transfections

African green monkey kidney cells (COS7) and human embryonic kidney 293T (HEK293T) cells were maintained in DMEM (Euroclone) supplemented with 10% fetal bovine serum (Euroclone), 2 mM L-glutamine (Euroclone) and penicillin/streptomycin (100 units/mL and 100 µg/mL respectively, Euroclone) at 37 °C with 5% CO_2_. Cells were transfected with Lipofectamine^TM^ 3000 (Life Technologies Incorporated).

Primary hippocampal cultures were prepared from embryonic day 17 (E17) mouse embryos considering the day of the vaginal plug as E0, as described previously [47], and plated on poly-L-lysine (Sigma-Aldrich, Sant Louis, MO 63103, USA) coated plates.

CDKL5 expression was abruptly silenced in primary hippocampal neurons at DIV11 through the transfection of a shCDKL5 targeting the sequence GCAGAGTCGGCACAGCTAT, using as negative control a shRNA against LacZ (shLacZ). Plasmid transfection was performed with Lipofectamine^TM^ 2000 transfection reagent (Life Technologies Incorporated).

### 4.4. Pharmacological Treatment

For treatment in vitro, 0.3 or 1 μM of PME or 0.1% DMSO (vehicle) was added to the primary cultures at DIV11, and neurons were harvested after 72 h. For treatment in vivo, mice received daily subcutaneous injections from PND60 to PND66 of 10 mg/kg of PME or sesame oil (vehicle) between 9:00–11:00 as described in Barbiero et al. [15]. For neuroanatomical experiments, mice were sacrificed 24 h after ended treatment and processed as described below. PME was suspended in sesame oil and freshly prepared each day.

### 4.5. Antibodies

The following primary antibodies were used in immunofluorescence and western blotting experiments: mouse anti-bassoon (Santa Cruz, sc-58509), rabbit anti-CDKL5 (Sigma, HPA002847), mouse anti-CDKL5 (Santa Cruz, sc-376314), rabbit anti-collybistin (SYSY, 261003), rabbit anti-GABA_A_R γ_2_ (SYSY, 224003), goat anti-GABA_A_R γ_2_ (Invitrogen, PA5-19299), rabbit anti-GAPDH (Sigma, G9545), mouse anti-gephyrin (Santa Cruz, sc-25311), anti-GFP (chicken, Molecular Probes, A10262), chicken anti-MAP2 (SYSY, 188006), mouse anti-c-myc (clone 9E10), rabbit anti-VGAT (SYSY, 131003). Secondary Alexa Fluor anti-rabbit, -mouse and -chicken were purchased from Abcam or Invitrogen. HRP-conjugated goat anti-mouse, goat anti-rabbit and donkey anti-goat secondary antibodies for western blottings were purchased from Jackson Immunoresearch.

### 4.6. Immunofluorescence

COS7 cells and hippocampal neurons: neurons were grown on poly-L-lysine (1 mg/mL; Sigma-Aldrich) coated coverslips (300 cells/mm^2^) until DIV14. After fixation in 4% formaldehyde (Pierce^TM^) with 4% sucrose (Sigma-Aldrich, Sant Louis, MO 63103, USA), cells were blocked in 1X PBS, 5% goat serum (Euroclone) and 0.2% Triton X-100. Surface exposed γ_2_ subunit-containing GABA_A_R were immunostained under non-permeabilising conditions with blocking in 1X PBS, 5% goat serum. Incubation with the primary antibody was performed overnight at 4 °C and with the secondary antibody for 1 h at room temperature. Slides were mounted with ProLong Gold antifade reagent (Life Technologies).

To quantify gephyrin, bassoon and VGAT puncta as well as surface expressed GABA_A_R γ_2_ along MAP2-positive dendrites, images were captured with a 60X objective coupled to an Olympus BX51 fluorescence microscope equipped with Retiga R1 (QImaging) CCD camera. The number of gephyrin, bassoon and VGAT puncta, along with the fluorescence intensity of GABA_A_R γ_2_ staining, were measured along 30 µm long segments (proximal part of secondary branches) using the software Fiji ImageJ (function: analyse particles or measure). Primary antibodies were used: bassoon, 1:50; CDKL5, 1:50; GABA_A_R γ_2_, 1:150; gephyrin, 1:50; GFP, 1:100; MAP2 chicken, 1:500; MAP2 rabbit and mouse, 1:1000; Myc, 1:200; VGAT, 1:1000.

Hippocampal slices: 24 h after treatment, mice were decapitated (upon dislocation) and brain hemispheres were rapidly excised and frozen in liquid nitrogen. Cryosections (30 μm) were cut and mounted onto coated slides (SuperFrost^®^ Plus, Thermo Scientific, 38116 Braunschweig, Germany) and stored at −80 °C. The samples were fixed in 2% paraformaldehyde (4 °C) for 90 s, rinsed thrice with 1X PBS and blocked for 1 h in blocking solution (0.05% goat serum, 3% Triton X-100 in 1X PBS). Upon incubation with anti-GABA_A_R γ_2_ antibody (1:1000) in a humid chamber overnight at 4 °C, the slices were incubated with the secondary antibody (Alexa Fluor goat anti-rabbit 488 nm) in blocking solution for 1 h at room temperature, rinsed thrice with 1X PBS and mounted with ProLong Gold antifade reagent (Life Technologies). As negative control, a sample was incubated with only the secondary antibody.

Images from the molecular layer of the dentate gyrus were acquired with a LEICA TCS SL confocal microscope (LEITZ; Leica Mycrosystems, Wetzlar, Germany) with objective 63X (NA 1.32; zoom factor = 8) and the pinhole set at 1 Airy unit. Four slices per animal were analysed, and the number of GABA_A_R γ_2_ clusters was quantified using the software Fiji ImageJ (plugin: analyse particles). Optimised threshold values and size filters were applied for all the images to identify GABA_A_R γ_2_ clusters. The number of GABA_A_R γ_2_ puncta was calculated in four identical sections for each slice (to have a mean of four separate zones of the dentate gyrus per single slice) and expressed per μm^2^.

### 4.7. Western Blotting and Immunoprecipitation

Primary hippocampal neurons were lysed in 3X Laemmli buffer, and samples were separated by 10% SDS-PAGE, transferred to nitrocellulose membranes and blocked in 5% non-fat milk in TBS-T (20 mM of Tris-HCl pH 7.4, 150 mM of NaCl, 0.2% Tween-20). Blots were incubated with primary antibodies overnight at 4 °C, washed in TBS-T and incubated with appropriate secondary antibodies for 1 h at room temperature. Blots were developed with protein detection system-ECL (Genespin) coupled to G:BOX Chemi Imaging System (Syngene). Densitometric expression analyses were performed using ImageJ software.

CDKL5 was immunoprecipitated from 400 μg of HEK293T cells or 1 mg of a mouse brain extract (PND20-30) lysed in lysis buffer [mM: 50 Tris-HCl pH 7.4, 150 NaCl, 1 EDTA, 1 EGTA, 1% Triton X-100, 1X protease inhibitor cocktail (PIC, Sigma-Aldrich, Sant Louis, MO 63103, USA) and 1X PhosSTOP (Roche)] and incubated overnight at 4 °C with 1 µg of anti-CDKL5 or unrelated IgGs as control. The immunocomplexes were precipitated with protein-G agarose (Life Technologies), washed several times with lysis buffer and analysed by SDS-PAGE and western blotting.

### 4.8. Biotinylation Assays

Biotinylation assays were performed according to previously described protocols [48,49] with slight modifications. Primary hippocampal neurons were plated in 6-well plates coated with 0.5 mg/mL poly-L-lysine (Sigma-Aldrich, 400,000 neurons/well) and used at DIV14. Neurons were washed twice with HBSS/Ca^2+^/Mg^2+^, followed by incubation with 0.5 mg/mL of Sulfo-NHS-SS-Biotin (Cyanagen). Quenching was performed using HBSS/Ca^2+^/Mg^2+^ supplemented with glycine (50 mM) and BSA (0.5%), after which cells were lysed with standard radio-immunoprecipitation assay (RIPA) buffer (mM: 50 Tris-HCl pH 7.4, 150 NaCl, 1 EDTA, 2 EGTA, 1% NP40, 0.1% SDS, 0.5% SDC, 1X PhosSTOP, 1X PIC). After correction for protein content using a BCA protein assay kit (Pierce^TM^), biotinylated proteins were purified on StreptAvidin UltraLink Resin (Pierce^TM^) and resolved by SDS-PAGE and western blotting. Surface expression was evaluated as the ratio between the biotinylated fraction (surface) and the total cell lysate normalised with GAPDH.

### 4.9. In Vitro Electrophysiological Recordings

GABA-mediated inhibitory postsynaptic currents in miniature (mIPSCs) were recorded using the patch-clamp technique in the whole cell voltage-clamp configuration in the presence of 1 µM tetrodotoxin (TTX, Tocris) to block the generation of action potentials. Recordings were obtained from primary hippocampal neurons at DIV14 (plated on poly-L-lysine coated coverslips at the density of 234 cells/mm^2^) using the Axopatch 200B amplifier and the pClamp-10 software (Axon Instruments). Recordings were performed in Krebs’-Ringer’s-HEPES (KRH) external solution (mM: 125 NaCl, 5 KCl, 1.2 MgSO_4_, 1.2 KH_2_PO_4_, 2 CaCl_2_, 6 glucose, 25 HEPES-NaOH pH 7.4). Recording pipettes were pulled from glass capillary (World Precision Instrument) using a two-stage puller (Narishige) and had tip resistances of 3–5 MΩ when filled with the intracellular solution (mM: 130 Cs-gluconate, 8 CsCl, 2 NaCl, 10 HEPES, 4 EGTA, 4 MgATP, 0.3 GTP pH 7.3). Voltage-clamp recordings were performed at holding potentials of +10 mV. The recorded traces were analysed using Clapfit-pClamp 10 software, after choosing an appropriate threshold. In particular, events that exceeded at least twice the standard deviation of the baseline noise were considered as mIPSCs and included in our analyses.

### 4.10. Statistical Analyses

All experiments and analyses were performed knowing the respective genotypes of the animals. Data were analysed with Prism software (GraphPad), and all values were expressed as the mean ± SEM. Data that were identified as significant outliers by the software were removed from the datasets. The significance of the western blotting and immunofluorescence results was evaluated using Unpaired Student’s *t*-test, One-way ANOVA followed by Tukey’s multiple comparisons test and Two-way ANOVA followed by Tukey’s multiple comparisons test. The significance of in vitro electrophysiological studies was evaluated by Mann Whitney U test, Kruskal-Wallis test followed by Dunn’s multiple comparisons test, One-way ANOVA followed by Tukey’s multiple comparisons test and Unpaired Student’s *t*-test. Probability values of *p* < 0.05 were considered as statistically significant.

## Data Availability

Not applicable.
